# Mechanisms that influence sex ratio variation in the invasive hymenopteran *Sirex noctilio* in South Africa

**DOI:** 10.1002/ece3.5305

**Published:** 2019-06-28

**Authors:** Joséphine Queffelec, Amy L. Wooding, Jaco M. Greeff, Jeffrey R. Garnas, Brett P. Hurley, Michael J. Wingfield, Bernard Slippers

**Affiliations:** ^1^ Department of Biochemistry, Genetics and Microbiology, Forestry and Agricultural Biotechnology Institute University of Pretoria Pretoria South Africa; ^2^ Department of Biochemistry, Genetics and Microbiology University of Pretoria Pretoria South Africa; ^3^ Department of Natural Resources and the Environment University of New Hampshire Durham New Hampshire; ^4^ Department of Zoology, Forestry and Agricultural Biotechnology Institute University of Pretoria Pretoria South Africa

**Keywords:** complementary sex determination, constrained sex allocation, invasive species, population genetics, sex ratio

## Abstract

*Sirex noctilio* is an economically important invasive pest of commercial pine forestry in the Southern Hemisphere. Newly established invasive populations of this woodwasp are characterized by highly male‐biased sex ratios that subsequently revert to those seen in the native range. This trend was not observed in the population of *S. noctilio* from the summer rainfall regions in South Africa, which remained highly male‐biased for almost a decade. The aim of this study was to determine the cause of this persistent male bias. As an explanation for this pattern, we test hypotheses related to mating success, female investment in male versus female offspring, and genetic diversity affecting diploid male production due to complementary sex determination. We found that 61% of females in a newly established *S. noctilio* population were mated. Microsatellite data analysis showed that populations of *S. noctilio* from the summer rainfall regions in South Africa are far less genetically diverse than those from the winter rainfall region, with mean Nei's unbiased gene diversity indexes of 0.056 and 0.273, respectively. These data also identified diploid males at low frequencies in both the winter (5%) and summer (2%) rainfall regions. The results suggest the presence of a complementary sex determination mechanism in *S. noctilio*, but imply that reduced genetic diversity is not the main driver of the male bias observed in the summer rainfall region. Among all the factors considered, selective investment in sons appears to have the most significant influence on male bias in *S. noctilio* populations. Why this investment remains different in frontier or early invasive populations is not clear but could be influenced by females laying unfertilized eggs to avoid diploid male production in populations with a high genetic relatedness.

## 1 INTRODUCTION

Fisher's principle states that “the sex ratio is in equilibrium when, in the population as a whole, the totals of effort spent producing the two sexes are equal” (Fisher, 1958; Hamilton, 1967). This is generally taken to mean that all populations will evolve toward a 1:1 sex ratio (Hamilton, 1967). However, in insects, sex ratios can vary due to several factors. These include local mate competition (Hamilton, 1967), host quality (Bono & Herbers, 2003; Charnov, 1982; Charnov & Skinner, 1984; Craig, Price, & Itami, 1992), genetic elements such as paternal sex ratio chromosomes (Werren & Stouthamer, 2003), reproductive parasites (Jiggins, Bentley, Majerus, & Hurst, 2001), and constrained sex allocation due to a low number of mating or a reduced sperm contribution from males (Godfray, 1990; Henter, 2004; Metzger, Bernstein, & Desouhant, 2008).

The order Hymenoptera (bees, wasps, and ants) is characterized by haplodiploidy where females develop from diploid, fertilized eggs and males from haploid, unfertilized eggs. This reproductive mode, also known as arrhenotoky, is possible through various sex determination systems, but the most commonly found within the Hymenoptera is complementary sex determination (CSD) (Asplen, Whitfield, De Boer, & Heimpel, 2009). Through this mechanism, individuals that are heterozygous at the *csd* locus/loci develop into females while homozygous or hemizygous individuals develop into males (Cook, 1993; Cook & Crozier, 1995; Whiting, 1943). This system can result in maladaptive male‐biased sex ratios, particularly under elevated levels of inbreeding which results in a surplus of diploid males (Van Wilgenburg, Driessen, & Beukeboom, 2006).

Introduced insect populations have typically undergone a population bottleneck and are sometimes characterized by low genetic diversity (Garnas et al., 2016). Inbreeding in small introduced populations that lack diversity at the *csd* locus tends to increase the production of homozygous males at the expense of female progeny (Collet et al., 2016; Gloag et al., 2016). Gloag et al. (2016) studied the dynamics of *csd* allele frequencies in a population of *Apis cerana* (Fabricius) (Hymenoptera: Apidae) after its introduction in Australia. The bottleneck experienced during introduction caused the *csd* alleles to have different frequencies with a few alleles dominating, shortly after the bee's arrival, resulting in the production of a large number of diploid males. After a few generations, rare alleles increased in frequency due to diversifying selection on the *csd* locus.

Additional introduction events, gene flow, and mutation can also lead to the appearance of new alleles. Gloag et al. (2016) modeled the resulting diploid male frequency in the population and observed that the frequency of diploid males gradually drops as frequency‐dependent selection favors rare *csd* alleles pushing allele frequencies toward parity. Repeated bottlenecks along the advancing front of an invasion are possible (Ochocki & Miller, 2017), especially when anthropogenic spread is important, which could lead to a pattern of male bias along the invasion edge. However, multiple introductions—whether from the center of existing infestations or from abroad—are likely to mitigate this effect over time (Garnas et al., 2016).

The woodwasp *Sirex noctilio* (Fabricius) (Hymenoptera: Siricidae) has been an extremely successful invader, spreading from its native range in Europe and North Africa to the non‐native pine plantations of the Southern Hemisphere, the natural pine forests of northeastern North America, and most recently to China (Li et al., 2015; Slippers, de Groot, & Wingfield, 2012). Invasive populations of *S. noctilio* in the Southern Hemisphere are characterized by an initial highly male‐biased sex ratios, with up to 32:1 males: females being recorded in Brazil (Iede, Penteado, & Schaitza, 1998), 20:1 in New Zealand (Zondag & Nuttall, 1977), 12:1 in South Africa (Hurley et al., 2008), and 16.5:1 in Tasmania (Taylor, 1981). These ratios tend to decrease with population age, and in established populations, reflect ratios of ~2:1 usually observed in the wasps’ native range (Spradbery & Kirk, 1978). This trend has been observed in the winter rainfall region of South Africa (i.e., Western Cape province). A sex ratio of 10.2:1 was observed in 1994 and gradually decreased to 5:1 in 1996 and to 3.3:1 in 2004 (Tribe & Cillié, 2004). In the summer rainfall regions of South Africa (i.e., KwaZulu‐Natal and Mpumalanga), the sex ratios have remained unusually high, with above 10 males:1 female for over 8 years.

The persistent high male bias observed in the populations of *S. noctilio* from the summer rainfall regions prompted an investigation into factors that are likely to influence the sex ratio in these populations. We tested two hypotheses: (a) Females in newly established populations of *S. noctilio* are under constrained sex allocation (experience low mating success), which causes increased production of haploid male offspring; (b) decreased genetic diversity in the population is acting on a CSD system, leading to high levels of homozygosity at the sex determination locus/loci and resulting in the production of diploid males and an increased male bias in these populations. The presence of CSD has not been demonstrated in *S. noctilio*. However, we chose to consider this hypothesis because of the high prevalence of this sex determination mechanism among Hymenoptera and its tendency to skew the sex ratio toward males.

## 2 MATERIALS AND METHODS

### 2.1 Sample collection for microsatellite analyses

Only male wasps were used for microsatellite analyses. They were obtained from the collections of the Forestry and Agricultural Biotechnology Institute (FABI) at the University of Pretoria, South Africa. The wasp collections were from trees felled annually in various pine plantations in South Africa for the purpose of screening emerging wasps for the presence of the parasitic nematode *Deladenus siricidicola (*Bedding) (Tylenchida: Neotylenchidae) (Verleur, Croft, & Germishuizen, 2017). Felled trees were sectioned and placed in emergence drums at different locations in the country. Emerging wasps were collected and sent to FABI where they were stored in ethanol. Sixty‐seven male wasps (collected during the November 2010 through February 2011 emergence period) from the Western Cape province and 77 from KwaZulu‐Natal were used. The two provinces are 1,200 km apart. Wasps were dissected to obtain thoracic tissue for DNA extraction.

### 2.2 DNA extraction and microsatellite screening

DNA was extracted using the *prep*GEM™ Insect kit DNA extraction kit (ZyGEM Corporation Ltd) following the manufacturers’ instructions. Samples were amplified at 13 microsatellite loci reported in Santana et al. (2009), using the QuantiTect^®^ Multiplex PCR Kit (Qiagen) and primers from Inqaba Biotec (Pretoria) (Santana et al., 2009). Amplification was performed using the manufacturer's instructions but modified such that all reactions were scaled down to a total volume of 8.2 µl. Cycling conditions were 95°C for 15 min followed by fifty cycles of 94°C for 1 min and 60°C for 1.5 min.

Amplified products were visualized using agarose gel electrophoresis on a 2% agarose gel. PCR products were loaded with 30× GelRed (Biotium). Visualized PCR products were analyzed by electrophoresis in an ABI PRISM 3100 Automated DNA Sequencer (Applied Biosystems). The data collected were analyzed using GeneMapper software (version 3.0; Applied Biosystems) for genotyping of all samples at the 13 loci. All allele calls made by the software were checked manually, and in cases where complete genotypes were not obtained, samples were excluded from further analyses.

### 2.3 Analysis of genetic diversity

A CSD locus has not been identified in *S. noctilio*. Consequently, neutral genetic diversity was calculated from microsatellite data sets. Nei's unbiased gene diversity index and allelic richness were used as indices of genetic diversity and calculated using the Poppr package (Kamvar, Tabima, & Grünwald, 2014) in R v.3.4.2 (R Core Team, 2017).

### 2.4 Determination of ploidy and statistical analysis

After genotyping the male wasps, we used heterozygosity at one or more loci to indicate diploidy, and homozygosity at all loci to indicate haploidy. The presence of diploid males was estimated from the frequency of heterozygous males, *e*, since a heterozygous male must be diploid. However, only males that are heterozygous at at least one locus can be detected using this approach and this detection limit, *d,* needs to be calculated and can be used to correct *e*.

$$e_{\text{corrected}}\;=\frac{e}{d}$$

We calculated *d* with *p*_i_ = the frequency of the *i*th allele and *j* indicating the *j*th locus.

$$d=\;1-\prod_{j}\sum_{i}p_{i}$$

### 2.5 Proportion of females experiencing constrained sex allocation

Constrained females are defined as those that have no sperm in their spermathecae (sperm storage organ; (Godfray, 1990)). Wasps were collected in 2012 from recently invaded pine plantations in the Mpumalanga province of South Africa, using black panel traps baited with a kairomone lure. The wasps were collected from three trap sites over a six week emergence period. Seventy‐five females were dissected, and their spermathecae were crushed on a slide using a scalpel blade and stained using bromophenol blue (Sigma‐Aldrich). The slides were viewed under a Zeiss Axioskop 2 plus light microscope at 10×, 20×, 40×, and 100× magnification and screened for the presence or absence of sperm, which in *S. noctilio* has a distinctive shape with “spikes” radiating from a central “hub” (Phillips, 1970).

### 2.6 Calculation of observed male:female ratios

Investment in haploid eggs (sons), where sex allocation is unconstrained by mating status, can influence the observed adult sex ratio. Therefore, a state‐space model was constructed. It allowed us to systematically vary the frequency of constrained females (*c*) and the proportional investment in unfertilized eggs among mated females (*r*). Two scenarios were considered. First, when CSD is absent in *S. noctilio*, the observed ratio of males to females (OSR_ca_) can be calculated as:

(1) $${\text{OSR}}_{\text{ca}}=\frac{c+(1-c)r}{\left(1-c)(1-r\right)}$$

Second, when CSD is present, more variables are required. Assume the proportion of diploid males that survive is *s* and the number of *csd* alleles in the population is *x*, all having a frequency of *x* ^−1^. If the fraction of haploid males is *m*_h_, the fraction of diploid males is *m_d,_* and the fraction of females is *f*, and we denote the fraction of males by *m* = *m*_h_ + *m*_d_, then we can write three recursion equations for f, *m*_h_, and *m*_d_ and solve for their equilibrium values (${\tilde{m}}_{h}$, ${\tilde{m}}_{d}$, and $\tilde{f}$) that are reached within a few generation (Data S1). Here, we assume that diploid males do not produce viable sperm. The ratio of observed males to females = (${\tilde{m}}_{h}$ + ${\tilde{m}}_{d}$)/$\tilde{f}$ can then be calculated as

(2) $${\text{OSR}}_{\text{cp}}=\frac{2\text{as}+bx+\sqrt{x\left(4\text{as}+b^{2}x\right)}}{2a\left(x-1\right)}$$

with a = (1 − c)(1 − *r*) and b = *r* + cr:

The fraction of males that are diploid is given by

(3) $$\frac{{\tilde{m}}_{d}}{\tilde{m}}=\frac{2\text{as}+bx-\sqrt{x\left(4\text{as}+b^{2}x\right)}}{2a\left(s-x\right)}$$

These equations made it possible to examine the effect of mating frequency, the initial investment in sons, the number of *csd* alleles, and the survival of diploid males on the sex ratio. We assumed that constrained and unconstrained females have the same realized fecundity, as observed in Opp and Prokopy (1986), Sousa and Spence (2000), and Metzger et al. (2008). Sex determination without CSD is essentially the same as an infinite number of CSD alleles. Hence, to give the highest bias possible under CSD with alleles at equal frequency, we considered two alleles.

## 3 RESULTS

### 3.1 Microsatellite scoring and analysis of genetic diversity

Various genetic diversity indices were obtained for 67 wasps from the Western Cape province and 77 wasps from KwaZulu‐Natal. Of the 13 loci analyzed, eight were polymorphic in the population from the Western Cape province and three were polymorphic in the population from KwaZulu‐Natal. Five of the 13 loci used were monomorphic in both populations. Unique alleles were found in the population from the Western Cape province, but no unique alleles were found in the population from KwaZulu‐Natal. The mean allelic richness and gene diversity indices were also lower in the KwaZulu‐Natal population than in the population from the Western Cape province (Table 1).

**Table 1:** Diversity indices for the Western Cape province (WC, *n* = 67) and KwaZulu‐Natal (KZN, *n* = 77) populations per locus

Locus	Nei's index per locus		Allelic richness	
	WC	KZN	WC	KZN
Sn231	0.502	0.000	2.000	1.000
Sn576	0.000	0.000	1.000	1.000
Sn350	0.498	0.000	7.000	1.000
SnA2	0.555	0.461	4.000	2.000
SnB4	0.000	0.000	1.000	1.000
Sn104	0.03	0.000	2.000	1.000
Sn177	0.000	0.000	1.000	1.000
Sn185	0.369	0.000	2.000	1.000
Sn525	0.000	0.000	1.000	1.000
Sn641	0.000	0.000	1.000	1.000
Sn90	0.435	0.000	4.000	1.000
SnA7	0.412	0.100	3.000	2.000
SnB2	0.633	0.167	3.000	2.000
Mean	0.273 (*SD* = 0.266)	0.056 (*SD* = 0.132)	2.461 (*SD* = 2.461)	1.230 (*SD* = 0.439)

### 3.2 Frequency of diploid males and unmated females

The detection limits were 0.99 for the Western Cape population and 0.59 for KwaZulu‐Natal. We found heterozygous males in both populations (3 of 67 in the Western Cape; 1 of 77 males in KwaZulu‐Natal). Hence, the corrected frequency of diploid males in the two populations was 5% in the Western Cape province and 2% in KwaZulu‐Natal. Of the 75 spermathecae obtained from females, 46 contained sperm, giving a proportion of constrained females of 38.7%.

### 3.3 Calculation of observed male:female ratios

**Figure 1:**
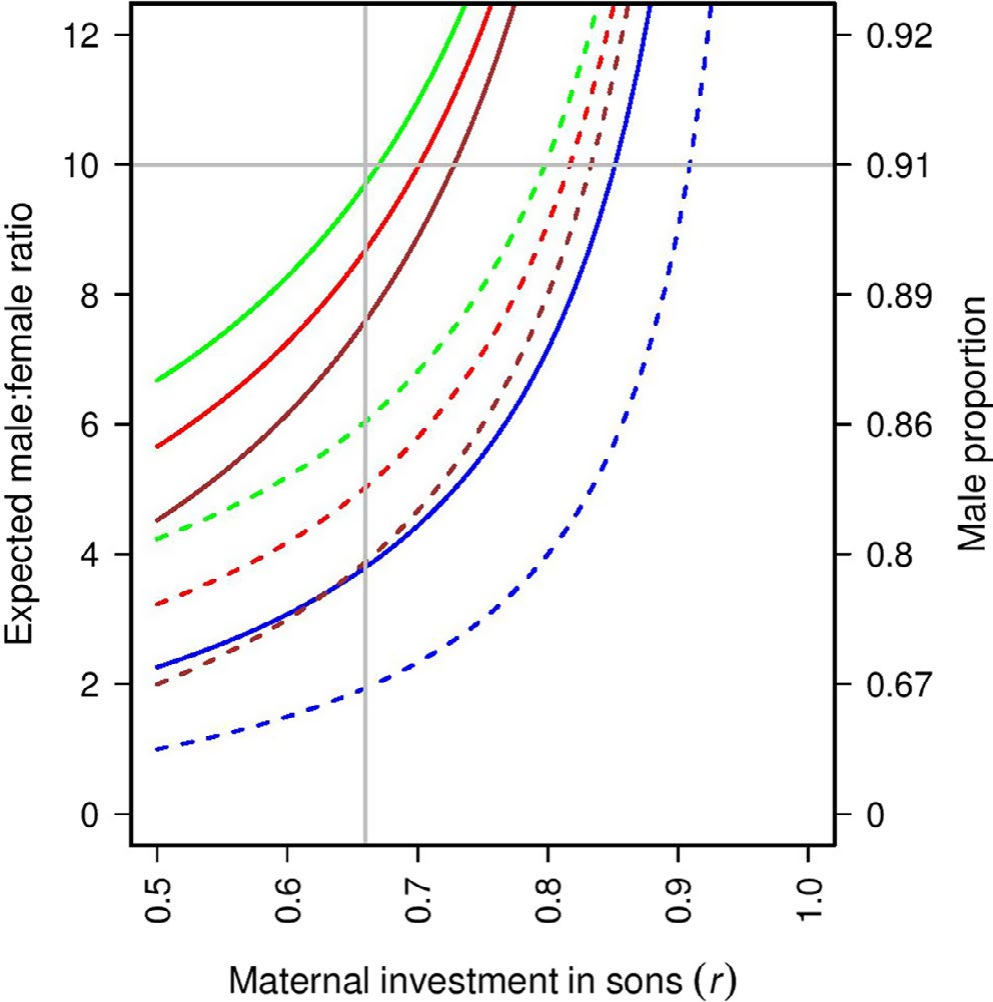
State‐space model showing the effects of the sex determination mechanism, the maternal proportional investment in sons (*r*) and sperm limitation (c) on male:female ratio. Blue lines correspond to the absence of CSD constraints (equivalent of Equation 1 with infinite alleles, x → ∞), whereas the other colors correspond to CSD with 2 csd alleles (x = 2; Equation 2). Dashed and solid lines represent the absence and observed level of constrained females, respectively (c = 0 and c = 0.387 in Equation 2); brown lines are for s = 0, red for s = 0.5, and green for s = 1. The horizontal gray line indicates the observed male:female ratio, and the vertical gray line indicates the typical investment in males in native populations

The state‐space model (Figure 1) showed that for all scenarios represented by different colors and line types, an increase in the maternal investment in sons results in an exponential increase in the observed male:female ratio. Consequently, to increase from 2:1 in a native population to 10:1 in an introduced population, females must invest more in sons in introduced populations compared with native populations. However, the number of *csd* alleles is usually higher in native populations and the frequency of constrained females might also be different between native and introduced populations. For CSD and non‐CSD sex determination systems, constrained females increase the sex ratio substantially (compare dashed and solid lines of each color). The observed proportion of males is lower when CSD is absent (compare blues lines to other colors). As the survival rate of diploid males increases, their frequency increases (Figure S1), as well as the sex ratio (Figure 1, compare brown, red, and green lines). In the case of a native population, to obtain an observed male:female ratio of 2:1, Figure 1 shows that the maternal investment in sons can vary between 0.5 and 0.667 depending on the presence (brown dashed line) and absence of CSD (blue dashed line). For an introduced population to produce a male:female ratio at 10:1 with a frequency of constrained females of 0.387 (solid lines), the maternal investment in sons varies between 0.671 and 0.852. The minimum maternal investment that could produce the observed skew is when CSD is present, leading to a proportion of diploid males with diploid males surviving, taking part in mating, but being sterile. The maximum maternal investment that can result in a 10:1 skew is when CSD is absent or the number of CSD alleles is large enough to prevent diploid male production.

## 4 DISCUSSION

Results of this study suggest that at the invasion front of *S. noctilio* in South Africa, sex ratios are correlated with a lack of genetic diversity. However, little support was found for the proposed mechanism linking low genetic diversity to the overproduction of diploid males. Mating frequency was sufficiently high in the populations from Mpumalanga to produce only a moderate male bias such as is seen in older invasive populations of *S. noctilio* (Tribe & Cillié, 2004). Together, these results suggest that female investment in male offspring is higher in the summer rainfall region of South Africa than in the native range or in the older invasive populations of *S. noctilio*.

**Figure 2:**
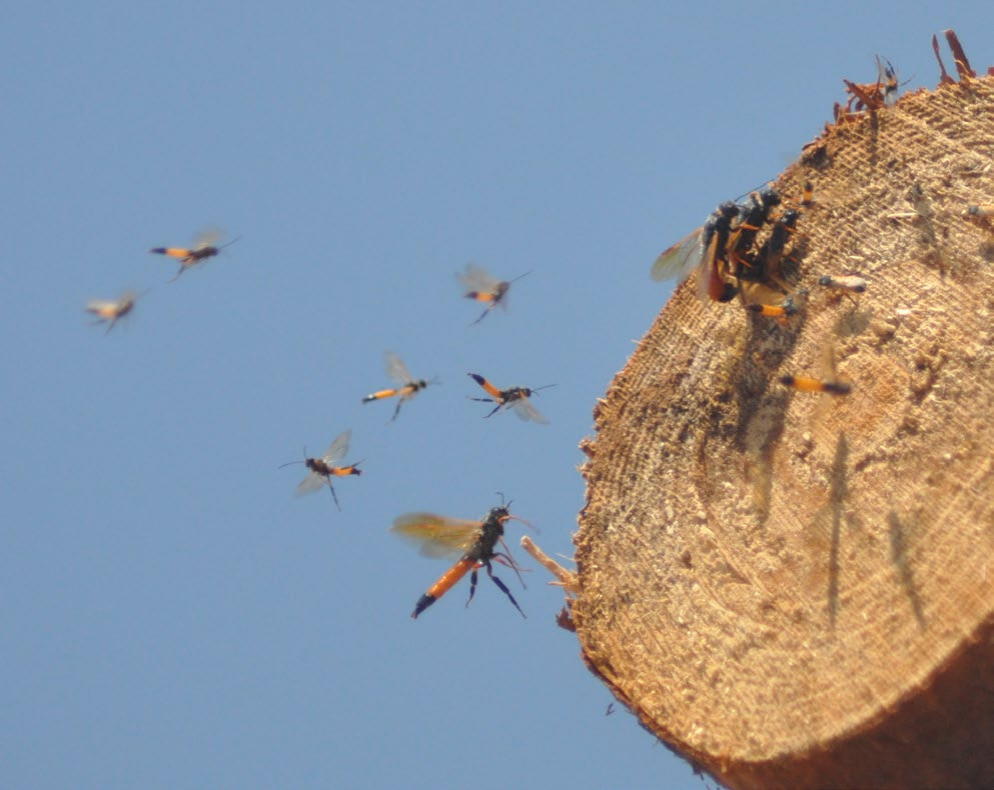
Female Sirex noctilio joining a lek of males and mating

Approximately 39% of the female *S. noctilio* considered in this study were subject to constrained sex allocation at the invasion front of the pest in Mpumalanga. Such constrained allocation explains in part the male bias of this population, but alone it is insufficient to explain the extreme male bias (2:1 vs. 10:1). Furthermore, this is a large proportion of constrained females when compared with other wild populations of hymenopteran insects that have a frequency of constrained females below 29% (Hardy & Godfray, 1990; West, Compton, Vincent, Herre, & Cook, 1998). This larger than expected proportion of constrained females could be an artifact of the timing of trap capture in that unmated females caught in traps may ultimately have mated. But this possibility would be negated by the fact that females have been observed to join leks of males (Figure 2), mate, and then disperse to other trees using host pheromones (Hurley, Garnas, & Cooperband, 2015). The only means to overcome this potential bias due to sampling would be to capture wasps as they oviposit. This would presumably need to be done after the mating swarm, which would be logistically challenging.

This study showed that at the time of sampling, the more strongly male‐biased populations of *S. noctilio* from KwaZulu‐Natal were considerably less genetically diverse than the populations from the Western Cape province. The low genetic diversity observed in KwaZulu‐Natal is not unusual, because this population was at the invasion front of *S. noctilio* in South Africa at the time of sampling. The population is hypothesized to have arisen from the movement of infested wood from the Western Cape province, because there is no continuous distribution of *Pinus* between this region and other regions where the woodwasp has been found (Hurley, Slippers, & Wingfield, 2007). For this reason, the population is expected to have experienced a founder effect associated with a newly established population (Dlugosch & Parker, 2008). The fact that no unique alleles were found in the population of *S. noctilio* from KwaZulu‐Natal supports previous work suggesting the Western Cape province as the source of the KwaZulu‐Natal populations (Hurley et al., 2007). It is relevant to also recognize that the high levels of genetic diversity observed in the population of *S. noctilio* from the Western Cape province, as well as the large number of unique alleles identified, support recent work showing that *S. noctilio* might have been introduced into South Africa more than once (Boissin et al., 2012).

The existence of diploid males in *S. noctilio* populations in South Africa supports the presence of a CSD mechanism in the woodwasp (Van Wilgenburg et al., 2006). The presence of CSD in *S. noctilio* is not unexpected as this mechanism is thought to be ancestral in the Hymenoptera (Asplen et al., 2009; de Boer, Kuijper, Heimpel, & Beukeboom, 2012; Schmieder, Colinet, & Poirié, 2012). However, some Hymenoptera that do not use CSD can still produce diploid males under certain circumstances. For example, the wasp *Nasonia virtripennis* (Walker) (Hymenoptera: Pteromalidae) does not use CSD, but a strain of triploid females can produce fertile diploid sons (Beukeboom, Kamping, & van de Zande, 2007). If diploid male production in *S. noctilio* arose from a similar mechanism to that in *N. vitripennis,* we would expect the occurrence of triploid females, but these have never been detected. Thus, while CSD is the most likely explanation for the existence of the diploid males in the populations of *S. noctilio*, it cannot be taken as irrefutable evidence for it.

Accurate estimation of the expected frequency of diploid males under CSD within a wild population is problematic without making assumptions about the number of matings in females, the number of *csd* alleles present, the survival rate of the diploid males, or the frequency of fertilization of eggs by females. The state‐space model designed in this study aimed at evaluating the strongest effect that diploid males could have on a sex ratio. To maximize the frequency of diploid males and their effect on the sex ratio, we made the assumption that females are monogamous but this does not reflect the reality (Caetano & Hajek, 2017); hence, it is not possible to compare the expected frequency of diploid males with what is observed in the field. However, it is possible to compare two populations with different genetic diversity. Given the hypothesis of CSD, and considering the lower genotypic diversity in KwaZulu‐Natal, a higher frequency of diploid males was expected in the population from KwaZulu‐Natal compared with the population from the Western Cape province. There are various possible hypotheses that could explain this situation. Firstly, while the microsatellite markers are useful to understand broad patterns of population variation, they carry the possibility of underestimating diploid males (e.g., if males homozygous at all 13 microsatellite loci are actually diploid). Also, neutral markers are generally unsuitable for detecting diversity at the CSD locus, which will be under strong frequency‐dependent selection.

The frequency of diploid males could have been influenced by their survival. Diploid males are not always viable and are generally rare in adult hymenopteran populations (Harpur, Sobhani, & Zayed, 2013). Along with errors that may arise in ploidy identification, a high mortality of diploid males before the imaginal stage could account for a low number of diploid males in our samples. This would also explain the absence of a significant difference between the frequency of diploid males between KwaZulu‐Natal and the Western Cape province.

The state‐space model developed in this study showed that the proportion of constrained (unmated) females observed in the summer rainfall region (~39%) does not provide the only possible explanation for the strong male bias in the sex ratio without a disproportionately high investment in males. The same conclusions can be drawn with and without the involvement of diploid males in mating. For example, the prediction corresponding to CSD and set to the observed mated frequency only matches the observed 10:1 male:female ratio when female investment in sons reaches ~67%. There is abundant published evidence that the frequency of fertilization in hymenopteran females can vary based on environmental conditions or cues or even population demographic or genetic parameters (West, 2009). Our data showed that female investment in male offspring has a greater influence on sex ratio than the frequency of constrained females over reasonable mating frequencies. In newly established populations, females may intentionally place unfertilized, male eggs in trees, though the reason for this is unclear. A further possibility is that survival from egg to adult differs by wasp gender and by condition within different parts of infested trees. Finally, sperm limitation in mated females cannot be ruled out (Boivin, 2013).

The apparent preference in wasps to place male eggs could reflect inbreeding avoidance and/or a mechanism to reduced diploid male production. In some hymenopteran species with CSD, females will avoid mating with males that are genetically similar at the sex determination locus (Harper, Bagley, Thompson, & Linnen, 2016; Metzger, Bernstein, Hoffmeister, & Desouhant, 2010; Ruf, Dorn, & Mazzi, 2012; Thiel, Weeda, De Boer, & Hoffmeister, 2013). Consequently, premating mechanisms linked to low diversity (e.g., avoidance of genetically similar mates) could also explain the higher than expected frequency of unmated females. Females might also avoid fertilization after mating with genetically similar males (Ruf et al., 2012). Whether this is the case in *S. noctilio* has yet to be determined.

The results of this study, while preliminary, provide a better understanding of the mechanisms that influence sex ratio in invasive populations of *S. noctilio* and their respective contribution. They also highlight the need for further exploration of sex determination in *S. noctilio*. Future studies directed at elucidating the genetic basis for sex determination in *S. noctilio* such as the identification of the sex determination locus or loci will lead to a better understanding of the male bias observed in newly established populations of the woodwasp. This study also highlighted the relevance of exploring preferential mating or fertilization (potentially due to genetic similarity) in populations of *S. noctilio*. Such studies will allow for an improved understanding of the extreme variation in sex ratios observed in invasive populations of *S. noctilio*.

## CONFLICT OF INTEREST

None declared.

## AUTHORS’ CONTRIBUTIONS

J. Queffelec contributed to the data analysis and interpretation and to the revision of the core conclusions of the study. A.L. Wooding carried out the acquisition of the data and contributed to the data analysis and interpretation and redaction of the work. Dr. J. Greeff, Dr. J.R. Garnas, and Dr. B.P. Hurley contributed to the study design, data analysis, and revision of the manuscript. Dr. M.J. Wingfield and Dr. B. Slippers contributed to the study design and the revision of the manuscript.

## Data Availability

The data underlying the main results of this study are openly available in Dryad at http://www.datadryad.org using the following https://doi.org/10.5061/dryad.f3p8j8g **.**
