# Supplementary material for: Mechanisms that influence sex ratio variation in the invasive hymenopteran Sirex noctilio in South Africa
Source: Ecol Evol. 2019 Jun 28;9(14):7966–73. doi: 10.1002/ece3.5305 (PMC6662311; doi:10.1002/ece3.5305)

We can briefly restate the definitions of the variables as:

*r* = proportion of haploid eggs the mother lays

*c* = fraction of females that are not mated

*s* = proportion of diploid males that survive

*x* = number of *csd* alleles

*f* = fraction of females

*m* = fraction of males

*m_h_* = fraction of males that are haploid

*m_d_* = fraction of males that are diploid

Three recursion equations can be obtained when these variables are combined in the model laid out in Table S1. Specifically, to get the relative amounts of females, diploid males and haploid males, we multiply columns 1, 2 and 3 or 4 or 5. To convert it to fractions we divide each by the sum of all three. If a prime denote frequency in the next generation it gives:

$$f^{'}=\left[ \left( 1-c \right)\left( m_{h}m^{-1} \right)\left( 1-r \right)\left( 1-x^{-1} \right) \right]/t$$

$$m_{h}^{'}=\left[ c+\left( 1-c \right)\left( \left( m_{h}m^{-1} \right)r+\left( m_{d}m^{-1} \right) \right) \right]/t$$

$$m_{d}^{'}=\left[ \left( 1-c \right)\left( m_{h}m^{-1} \right)\left( 1-r \right)sx^{-1} \right]/t$$

with *t* = sum of square brackets, which is $1-((1-c)m_{h}m^{-1}(1-r)(1-s)x^{-1})$.

By setting primes equal to current values and remembering that $m_{h}+m_{d}+f=1$, we can solve for the equilibrium values - $\tilde{m}_{h}$, $\tilde{m}_{d}$ and $\tilde{f}$, which can be combined to obtain equations 2 and 3 from main text.

**Supplementary table 1:** Potential mating and the resulting offspring for the model.

| Potential matings | |  |  | Resulting offspring | | |
| --- | --- | --- | --- | --- | --- | --- |
| Female mated or not | Frequency of kind of male mated |  |  | female | haploid male | diploid male |
| 1-*c* | *m*_h_*m*^-1^  *m*_d_*m*^-1^ |  |  | (1-*r*)(1-*x*^-1^) | *r*  1 | (1-*r*)*sx*^-1^ |
| *c* |  |  |  |  | 1 |  |

**Supplementary figure 1:** The fraction of diploid males predicted by [Eq 3] with the presence of CSD with *x* = 2, given maternal investment in sons (*r*), fraction of constrained mothers (dashed lines: *c* = 0; solid lines: c=0.387) and the survival of diploid males (line colours as in Fig 1, brown: *s* = 0, red: *s* = 0.5 and green: *s* = 1).


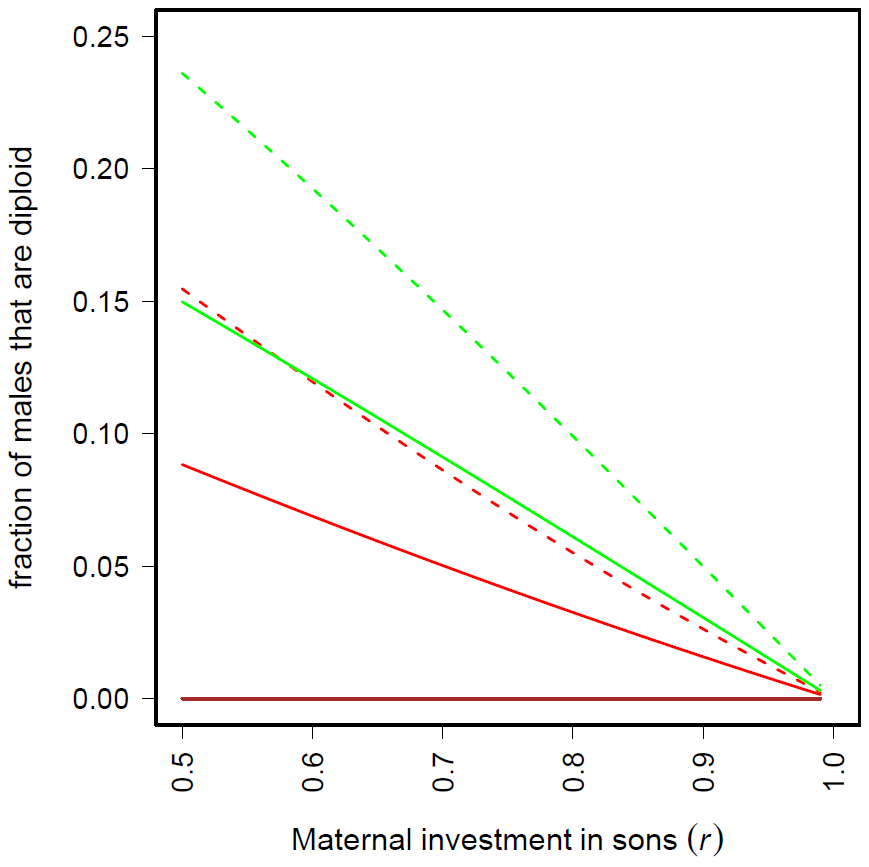

Supplement: Supplementary file 1 [file ECE3-9-7966-s001.docx]
